# Audience Effect Alters Male Mating Preferences in Zebra Finches (*Taeniopygia guttata*)

**DOI:** 10.1371/journal.pone.0043697

**Published:** 2012-08-20

**Authors:** Frédérique Dubois, Alexandra Belzile

**Affiliations:** Département de Sciences Biologiques, Université de Montréal, Montréal, Québec, Canada; University of Saint-Etienne, France

## Abstract

The social environment of animals strongly influences the mating preferences of both the choosing and the observing individuals. Notably, there is recent evidence that polygamous males decrease their selectivity when being observed by competitors in order to direct their rivals’ attention away from their true interest and, consequently, reduce sperm competition risk. Yet, other mechanisms, whose importance remains unexplored, could induce similar effects. In monogamous species with mutual choice, particularly, if males adjust their selectivity according to the risk of being rejected by their preferred mate, they should as well become less selective when potential rivals are present. Here, we investigated whether the presence of bystanders modifies male mating preferences when the risk of sperm competition is low, by carrying out mate-choice experiments with male zebra finches (*Taeniopygia guttata*) whose preferences for two females were measured twice: with and without an audience. We found that the presence of potential rivals had no effect on the males’ choosiness. However, with an audience, they spent more time with the female that was considered as the less attractive one in the control condition. These findings support the hypothesis that monogamous males alter their mate choice decisions in the presence of a male audience to reduce the risk of remaining unpaired. Thus, our results indicate that several explanations can account for the changes in male preferences due to the presence of competitors and highlight the importance of assessing the relative role of each mechanism potentially involved, to be able to make conclusions about the effect of an audience on signal evolution.

## Introduction

The social context in which mate choice occurs can strongly influence the mating preferences of both the choosing and the observing (by-standing) individuals. For instance, a large number of studies investigating the role of social environment on the expression of mating preferences have demonstrated that males and females can acquire preferences for particular mates, by observing and copying the mate choice of same-sex conspecifics (see reviews [1]–[3]). Also, recent findings have reported that the mating preferences of the choosing individuals can be affected as well by the presence of an audience [4]–[6]. The most frequently invoked explanation for why males should modify their mating preferences in the presence of potential rivals is that they have to adjust their decisions according to the risk of sperm competition to ensure their fertilization success [5]–[8]. Support for this finding is that polygamous Atlantic molly (*Poecilia mexicana*) males both reduce the expression of their mating preferences (thereby providing less information about their preferences) and increase their preferences towards the initially non-preferred female (thereby leading their rivals away from their preferred mate), when a male audience is present (e.g. [5], [9]).

Up to now, the effect of an audience on male mating preferences has been investigated almost only in species where the risk of sperm competition is high. However, other mechanisms, whose importance remains unexplored, could also be responsible for changes in male mating decisions in response to the presence of audience males. In species with mutual mate choice, in particular, if males adjust their mate selectivity according to the risk of being rejected by their preferred mate or to their risk of remaining unpaired, then they should become less selective in the presence of male competitors, especially if audience males are more attractive to females. This prediction arises because unattractive males, when both sexes are selective in their choice, have almost no chance of reproducing with an attractive female, unless she has no other available option. Therefore, males should express their ideal mate preference only when they have no competitors, and consequently no risk of remaining unpaired. Conversely, when rivals are present and females consequently can choose among different potential partners of varying quality, males should adjust their preferences according to their mate-getting ability and then express their realized preference [10], [11]. Thus, according to this mechanism, the presence of a male audience should also lead to reduced male selectivity.

Several lines of evidence support the possible importance of this mechanism in species with mutual mate choice. First, recent findings have demonstrated that female zebra finches (*Taeniopygia guttata*) adjust their selectivity in relation to circumstances, and notably based on their own condition or mate-getting ability [11], [12]. Second, an effect of an audience on male mating behaviour has been shown in male zebra finches that respond more to their partner’s voice when in the presence of a mated pair [13]. Whether and how the presence of a male audience influences male mating decisions in species where the risk of sperm competition is low, however, has not been investigated yet. To address this question, we carried out binary mate-choice experiments with male zebra finches whose preference for two stimulus females were measured twice: with and without a male audience. The zebra finch is well-suited for this study because the birds breed in dense colonies [14]. Thus, a large number of individuals choose a mate simultaneously and the risk that bystanders are present while males make their decision, consequently, is high. Furthermore, there is accumulating evidence that the intensity of sperm competition is low in this socially monogamous species [15]–[17]. Finally, both sexes invest in parental care and hence are selective in their choice [18], [19]. Accordingly, several studies have demonstrated that females choose mates based on aesthetic traits, such as bill colour [20] that reflect male quality, while males prefer females with a higher fecundity [21], [22]. So, despite the fact that males and females use different criteria, both sexes participate in mate choice. For all these reasons, we would expect males to modify their mating preferences when potential rivals are present not to provide their rivals with misleading information, but rather to diminish the probability of being rejected by their preferred mate. We then predict that the presence of potential rivals in the zebra finch should decrease male selectivity but that the expression of male mating preferences (i.e. the relative choosing time) should be unaffected by a male audience.

## Materials and Methods

### Ethics Statement

The experiments described in this study were approved by the Animal Care Committee of the Université de Montréal (animal care permit #09–034) and conformed to all guidelines of the Canadian Council on Animal Care.

### Animals and Housing Conditions

We used 26 (20 males and 6 females) commercially purchased adult zebra finches obtained from a local breeder (Exotic Wings & Pet Things, St Clements, Ontario, Canada) ) ensuring that males and females were unrelated and had never been in contact before. All the birds were individually tagged with numbered plastic leg bands. Outside the test trials, they were housed in cages (38 cm×38 cm×48 cm) in same-sex groups of two to five birds, and kept on 13∶11 h light: dark photoperiod at a constant temperature of 23°C with an *ad libitum* access to seeds and water.

### Measures of Mating Preferences

**Figure 1:**
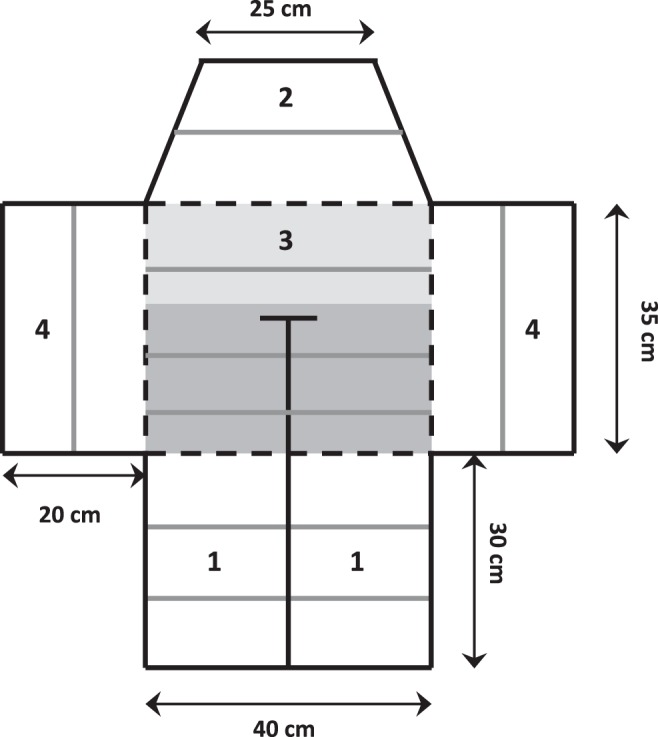
Top view of the binary mate-choice apparatus with: the female compartment (1), the end compartment (2), the choice compartment (3), and the observation compartments (4). The grey lines represent the perches while the black lines correspond to the partitions that were either opaque (full lines) or clear (dashed lines). The choice compartment (3) was divided into the neutral zone (pale grey) and the choice zone (dark grey).

We measured mating preferences with a classical binary choice apparatus (Figure 1) that comprised: 1) the female compartment that was divided into two identical chambers, each housing a single female, 2) the end compartment where the focal male could see both stimulus females simultaneously, 3) the choice compartment where it could see only one female at a time, and 4) two observation compartments where the audience males could observe the choice of the focal individual. Before the beginning of the experiments, all individuals were familiarized with the apparatus. Then focal males were tested under two experimental conditions: once with a male audience (i.e. with two males that were placed in the observation compartments) and once without an audience. Ten males were tested first with a male audience and then in the control condition (i.e. without a male audience), while the other males experienced the two conditions in the opposite order. Each focal male was tested during four consecutive days, because we estimated its preference twice in each condition, switching the position of the two stimulus females from one day to the next, to control for any side bias. For a given male, we used the same randomly chosen pair of females for the four preference tests, while the two males that served as rivals in the male audience condition were randomly chosen among the remaining individuals. All males, therefore, were used both as focal subjects and rivals.

The procedure we used to conduct a preference test consisted of two periods of 1 h each: during the first period, the focal male was confined to the end compartment, from which it could observe the two stimulus females. Then it was introduced into the choice compartment, and we measured the time spent in the neutral and choice zones as well as its preference for one or the other female as the time spent on the choice perches in front of each of them (Figure 1). We used this simple produce to measure male preferences, because the time spent in front of potential mates is correlated with actual mate choice in zebra finch [23], [24] and individuals are consistent in their choices when presented twice with the same set of mates [25]. Accordingly, we found that the time spent by males in front of each stimulus female was highly repeatable from one trial to the next (*r* = 0.594, *P* = 0.024), when we excluded from the analyses the two males that had a side bias.

### Data Analyses

We used general linear models (ANOVA) for repeated measures to test whether the males’ choosiness (i.e. the relative time spent in the choice zone) or their mating preferences (i.e. the percent of choosing time spent in front of each female) differed between the two conditions and depending on the order of treatments. Treatment was entered as a within-subject factor and the order of treatments was entered as a between-subject factor. Only males that had no side bias were included in the analyses, and all statistical analyses were performed with SPSS 16.0 for PC.

## Results

**Figure 2:**
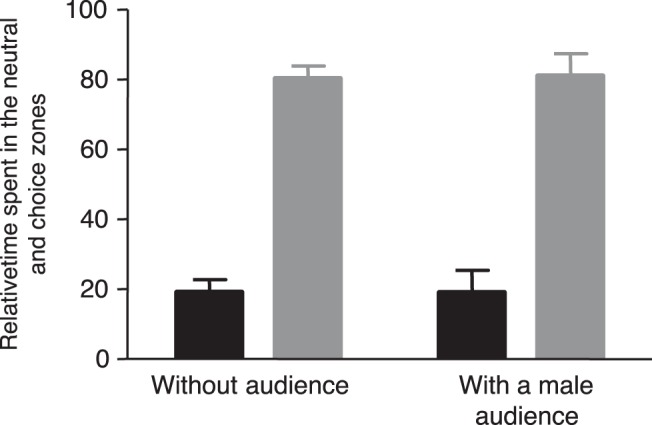
Mean (± SEM) percent of time spent by focal males in the neutral zone (black bars) and in the choice zone (grey bars) of the apparatus in both experimental conditions (i.e. with and without a male audience).

**Figure 3:**
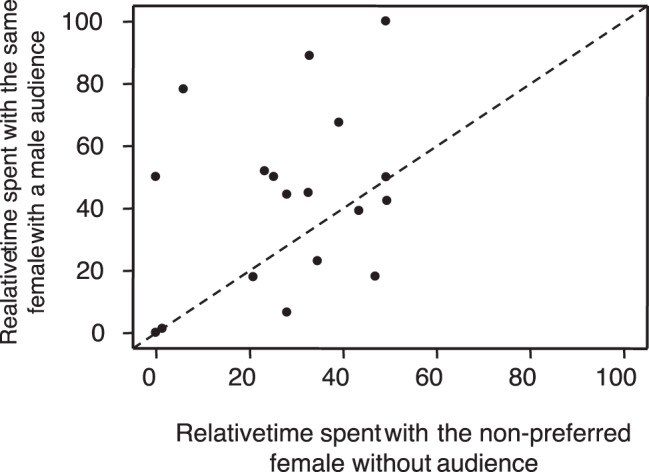
Influence of male audience on the time spent by the focal males with the female that was considered as the less attractive one in the control condition. Points above or below the dotted grey line represent respectively males that either decreased or increased their mating preference for the female that they considered the less attractive one, when there was no male audience.

The presence of a male audience had no effect on the time spent by focal individuals in the choice zone (mean ±SE percent of time in the choice zone: without audience: 80.32%±3.56, with a male audience: 81.00%±6.37; ANOVA: F_1,16_ = 0.007, *P* = 0.934; Figure 2) and there was no interaction between the order of treatments and the condition (ANOVA: F_1,16_ = 0.374, *P* = 0.549). Thus, our results indicate that males’ choosiness was not affected by the presence of rivals. Conversely, their mating preferences for each of the two stimulus females changed when there was a male audience. More precisely, we found that the relative time spent in front of the female that was considered as the less attractive in the control condition significantly increased when rivals were present (percent of time spent with the less preferred female: without audience: 28.34%±4.05, with a male audience: 42.99%±6.77; ANOVA: F_1,16_ = 4.543, *P* = 0.049; Figure 3). This effect was independent on the order in which the males experienced the two conditions, as revealed by the non significant interaction between the condition and the order of treatments (F_1,16_ = 0.033, *P* = 0.858).

## Discussion

Audience effects are increasingly recognized as an important aspect of intra-specific communication and have been demonstrated to play a crucial role in many contexts [26]. In particular, several studies have demonstrated that the presence of individuals other than those involved in the signalling interaction may change the signaller’s behaviour and affect signal evolution in both a male-male or male-female interaction. In Siamese fighting fish (*Betta spendens*), for instance, males display differently, during a male-male aggressive interaction, regarding on whether the audience is a male or a female [27], [28]. More precisely, when the audience is a female, they increase the intensity of conspicuous displays that can be used in communication with both males and females but decrease aggressive displays that are solely directed to males [27], [28]. Also, a number of experiments with monogamous species indicate that males modify their behaviour during a male-female interaction when in the presence of an audience [13], [29], [30]. For example, during pair interactions, male zebra finches respond more to their partner’s voice if a pair is in audience [13], while both budgerigar (*Melopsittacus undulates*) and canary (*Serinus canaria*) males reduce their extra-pair courtships in the presence of their partner [29], [30]. Our study provides additional evidence that audience effects are important in monogamous species and demonstrates for the first time that the presence of potential rivals may affect male mating decisions as well. Indeed, we found that focal males, with a male audience spent significantly more time with the female that was considered as the less attractive one in the control condition. A similar change in male mating preferences in the presence of competitors was reported by Plath and collaborators for polygamous molly males [5], [31]. In our study, however, the observed change in mate choice decisions was probably not a strategy to reduce the risk of sperm competition by providing no information or misleading information [31]. Instead, zebra finch males very likely have adjusted their preferences according to their mate-getting ability to reduce the risk of being rejected by their preferred female. This explanation is the most plausible for two reasons. First, the potential for sperm competition is low in this species, not only because extra-pair copulations are rare [15] but also because there is no evidence that males, contrary to females [32], [33], copy the choice of others [34]. Second, supporting our expectations, we found that the presence of bystanders influenced the relative time spent by focal males in front of each stimulus females but not their choosing time.

Thus, our findings support the hypothesis that different mechanisms can provoke a decrease in male mating preferences in the presence of competitors, indicating that the observed behavioural changes do not necessarily occur at the expense of the audience males. Previous studies have identified other explanations that could account for this pattern [5], highlighting the importance of assessing the relative role of each mechanism potentially involved, to be able to make conclusions about the impact of the audience on signal evolution. One way to do that would consist in examining differences in the extent to which individuals are affected by the presence of an audience. Indeed, there is theoretical and experimental evidence that bystanders differ in the importance they give to public [35], [36] and private information when their make their own decisions. This arises because the costs and benefits associated with public information use vary among individuals, depending on their age, quality or previous experience. For instance, mate-choice copying can be beneficial only if it provides individuals with more reliable information about the quality of potential partners and an animal’s propensity to copy, therefore, should depend on its ability to distinguish mate qualities [37], [38]. Supporting this prediction, Dugatkin and Godin [39] have shown that young female guppies (*Poecilia reticulata*) with poor mate-assessment ability copy the mate choice of experienced females, while old females are not influenced by the mating decisions of younger individuals. For similar reasons, we would expect that not all observed individuals within a given population will be equally affected by the presence of bystanders, and so whatever the mechanism causing the changes in mate choice decisions. Indeed, if males adjust their preferences in relation to their mate-getting ability, we would expect attractive males to be less affected by the presence of an audience. Conversely, if males modify their mating preferences to deceive their rivals and hence reduce sperm competition risk, the effect should be stronger for experienced males who have a greater risk of being imitated, compared to younger individuals whose mating decisions are less reliable. Exploring individual differences, therefore, might be useful for discriminating among alternative hypotheses, and hence for evaluating the importance of the observed effects on signal evolution.

In conclusion, results from our study indicate that the presence of a male audience can induce a decrease in male mating preferences, even under low risk of sperm competition. This suggests that audience effects on mate choice decisions would be more widespread than initially thought, as they would not be restricted to species with a high potential for sperm competition but can occur whenever mate choice is bidirectional. Furthermore, as the costs of being observed are likely to depend on individual characteristics, we recommend analyzing differences in the extent to which individuals are affected by the presence of an audience in further studies to improve our understanding of the dynamics of signalling interactions.
